# Supplementary material for: Dynamic Changes in Non-Invasive Markers of Liver Fibrosis Are Predictors of Liver Events after SVR in HCV Patients
Source: Viruses. 2023 May 26;15(6):1251. doi: 10.3390/v15061251 (PMC10302896; doi:10.3390/v15061251)
Supplement: Supplementary file 1 [file viruses-15-01251-s001.zip › viruses-2384822-supplementary.pdf]

Supplementary Materials:

Table S1. Baseline characteristics of the patients who developed or did not develop decompensated portal hypertension decompensation.

		No PHD n = 289 (90%)		PHD n = 32 (10%)		
**Characteristics**		**N (%) / Me**	**CI (95%) / P_{25}; P_{75}**	**N (%) / Me**	**CI (95%) / P_{25}; P_{75}**	p
**Sex**	*Male*	199 (689)	63.4; 74.2	22 (68.8)	51.8; 85.7	0.567
	Female	90 (31.1)	25.8; 36.5	10 (31.3)	14.3; 48.2	
**Alcohol user**	*Yes*	42 (14.6)	10.5; 18.8	6 (18.8)	4.5; 33.1	0.157
	Ex	62 (21.6)	16.8; 26.4	11 (34.4)	17.0; 51.8	
**IDUs**	*Yes*	5 (1.8)	0.2; 3.3	0 (0)	-	0.696
	Ex-IDUs	70 (24.6)	19.5; 29.6	7 (21.9)	6.7; 37.0	
**HT**		114 (39.4)	33.8; 45.1	12 (37.5)	19.8; 55.2	0.495
**DM**		61 (21.1)	16.4; 25.8	8 (25.0)	9.1; 40.9	0.377
**DL**		21 (7.3)	4.3; 10.3	0 (0)	-	0.102
**CKD**		12 (4.2)	1.8; 6.5	2 (6.3)	0; 15.1	0.416
**HD**		37 (12.8)	8.9; 16.7	6 (18.8)	4.5; 33.1	0.244
**Genotype**	*1*	228 (78.9)	74.2; 83.6	21 (65.6)	48.2; 83.0	0.752
	2	4 (1.4)	0; 2.7	0	-	
	3	37 (12.8)	8.9; 16.7	7 (21.9)	6.7; 37.0	
	4	20 (6.9)	4.0; 9.9	4 (12.5)	0.4; 24.6	
**Child-Pugh**	*A5*	248 (87.9)	84.1; 91.8	15 (46.9)	26.6; 63.7	<0.001
	A6	11 (3.9)	1.6; 6.2	4 (12.5)	0.4; 25.4	
	B7	17 (6.0)	3.2; 8.8	11 (34.4)	17.6; 53.3	
	B8	6 (2.1)	0.4; 3.8	2 (6.3)	0; 15.6	
	A	259 (91.8)	88.6; 95.1	19 (59.4)	39.7; 76.5	<0.001
	B	23 (8.2)	4.5; 11.4	13 (40.6)	23.5; 60.3	
**MELD**		7	(7; 7)	8	(7; 10.8)	<0.001

**TE (kPa)**		15.1	(11.8; 22.3)	25	(17.7;36.7)	0.003
**FIB-4**		2.6	(1.7; 4.8)	6.1	(4.3; 10.1)	<0.001
**APRI**		1.1	(0.6; 2.2)	2.2	(1.4; 4.3)	<0.001
**Previous decompensation**		20 (6.9)	4; 10	13 (40.9)	23; 59	<0.001

APRI: Aspartate-to-platelet ratio index; CI: Confidence interval; CKD: Chronic kidney disease; DL: Dyslipidemia; DM: Diabetes mellitus; PHD: Portal hypertension decompensation; HD: Heart Disease; HT: Hypertension; IDUs: Intravenous drug users; Me: Median; N: Number of patients; TE: Transient elastography.

Table S2. Factors associated with risk of developing portal hypertension decompensation and HCC (univariate analysis).

		Portal hypertension decompensation			HCC		
**Characteristics**		*HR*	**CI (95%)**	*p*	*HR*	**CI (95%)**	*p*
**Age**		-	-	-	1.06	0.99; 1.11	0.055
**Alcohol user**	*No*<br>*Yes*	1<br>1.40	0.57; 3.41	0.459	-	-	-
**Genotype**	*Others*<br>*GT 3*	-	-	-	1<br>5.75	1.78; 18.64	0.003
**DM**	*No*<br>*Yes*	-	-	-	1<br>3.89	1.25; 12.08	0.019
**DL**	*No*<br>*Yes*	1<br>0.45	0.00; 21.45	0.324	-	-	-
**Child-Pugh**	*A*<br>*B*	1<br>7.52	3.66; 15.46	<0.001	1<br>12.65	3.83; 41.79	<0.001
**Previous decompensation**	*No*<br>*Yes*	1<br>8.36	4.11; 16.99	<0.001	-	-	-
**MELD**		1.04	0.99; 1.07	0.057	1.04	0.99; 1.09	0.158
**TE (kPa)**		1.03	0.99; 1.06	0.097	-	-	-
**FIB-4**		1.12	1.07; 1.17	<0.001	1.12	1.04; 1.21	0.003
**APRI**		1.20	1.09; 1.31	<0.001	1.18	1.00; 1.39	0.050
**FIB-4 (1 year)**		1.44	1.29; 1.59	<0.001	1.29	1.07; 1.56	0.008
**APRI (1 year)**		1.09	0.99; 1.19	0.053	1.06	0.86; 1.31	0.605
**FIB-4 (2 years)**		1.51	1.33; 1.71	<0.001	1.40	1.18; 1.65	<0.001
**APRI (2 years)**		4.17	2.82; 6.17	<0.001	2.61	1.50; 4.53	0.001

APRI: Aspartate-to-platelet ratio index; CI: Confidence interval; DL: Dyslipidemia; DM: Diabetes mellitus; HR: Hazard Ratio; TE: Transient elastography.

Table S3. Baseline characteristics of the patients who developed or did not develop HCC.

		No HCC *n* = 309 (96.3%)		HCC *n* = 12 (3.7%)		
**Characteristics**		**N (%) / Me**	**CI (95%) / P_{25}; P_{75}**	**N (%) / Me**	**CI (95%) / P_{25}; P_{75}**	***p***
**Sex**	*Male*	212 (68.6)	63.4; 73.8	9 (75.0)	46.3; 100	0.455
	Female	97 (31.4)	26.2; 36.6	3 (25.0)	0; 53.7	
**Alcohol user**	*Yes*	46 (15.0)	11.0; 19.0	2 (16.7)	0; 41.4	0.635
	Ex	69 (22.5)	17.8; 27.2	4 (33.3)	2.1; 64.6	
**IDUs**	*Yes*	5 (1.6)	0.2; 3.1	0	-	0.364
	Ex-UDVP	76 (24.9)	20.0; 29.8	1 (8.3)	0; 26.7	
**HT**		122 (39.5)	34.0; 45.0	4 (33.3)	2.1; 64.6	0.458
**DM**		63 (20.5)	15.9; 24.9	6 (50.0)	16.8; 83.2	0.025
**DL**		20 (6.5)	3.7; 9.2	1 (8.3)	0; 26.7	0.562
**CKD**		14 (4.5)	2.2; 6.9	0 (0)	-	0.580
**HD**		41 (13.4)	9.5; 17.1	2 (16.8)	0; 41.4	0.495
**Genotype**	*1*	242 (78.3)	73.7; 82.9	7 (58.3)	25.6; 91.1	0.032
	2	4 (1.3)	0.03; 2.6	0	-	
	3	39 (12.6)	8.9; 16.3	5 (41.7)	9.0; 74.4	
	4	24 (7.8)	4.8; 10.8	0	-	
**Child-Pugh**	*A5*	257 (85.1)	81.1; 89.1	6 (50.0)	10.4; 80.5	<0.001
	A6	15 (5.0)	2.5; 7.4	0	-	
	B7	24 (7.9)	4.9; 11.0	4 (33.3)	2.5; 70.3	
	B8	6 (2.0)	0.4; 3.6	2 (16.7)	0; 45.4	
	A	272 (90.1)	86.7; 93.5	6 (50.0)	10.4; 80.5	<0.001
	B	30 (9.9)	6.5; 13.3	6 (50.0)	19.5; 89.6	

**MELD**		7	(7; 7.5)	7.5	(7.0; 12.8)	0.097
**FIB-4 baseline**		2.8	(1.7; 5.3)	5.4	(3.4; 9.6)	0.011
**APRI baseline**		1.3	(0.6; 2.2)	2.1	(0.8; 5.2)	0.152
**Previous decompensation**		29 (9.4)	6; 13	2 (16.7)	2; 65	0.340

APRI: Aspartate-to-platelet ratio index; CI: Confidence interval; CKD: Chronic kidney disease; DL: Dyslipidemia; DM: Diabetes mellitus; HCC: Hepatocellular carcinoma; HD: Heart disease; HT: Hypertension; IDUs: Intravenous drug users; Me: Median; N: Number of patients; TE: Transient elastography.
